# Coupled non-Hermitian skin effect with exceptional points

**DOI:** 10.1038/s41377-025-02006-6

**Published:** 2025-09-23

**Authors:** Guo-Huai Wang, Ran Tao, Zhen-Nan Tian, Qi-Dai Chen, Xu-Lin Zhang

**Affiliations:** State Key Laboratory of Integrated Optoelectronics, JLU Region, College of Electronic Science and Engineering, Jilin University, Changchun, 130012 China

**Keywords:** Integrated optics, Photonic crystals, Photonic devices

## Abstract

Non-Hermitian systems exhibit two unique hallmarks: exceptional points (EPs) and non-Hermitian skin effect (NHSE). The EP arises from the interplay of multiple energy levels, marked by degeneracy in eigenvalue spectra, while the NHSE is associated with the localization feature of eigenfunctions. Due to their different origins and consequences, the interplay between the two hallmarks has drawn considerable interest. Here, we propose the concept of coupled NHSE, i.e., two non-Hermitian systems with independent NHSE are coupled together. We find that by introducing non-Hermitian losses with special symmetry, multiple pairs of EPs can appear, greatly compressing the eigenvalue spectrum and accelerating the breakdown of the coupled NHSE. In contrast, the attenuation of coupled NHSE is significantly alleviated in systems without EPs. In this sense, the EP can act as a degree of freedom to tune the NHSE and govern the non-Hermitian dynamics. The proposed concept is experimentally realized in photonic lattices with artificial gauge fields, which will bridge these two significant concepts and open avenues for non-Hermitian applications simultaneously associated with them.

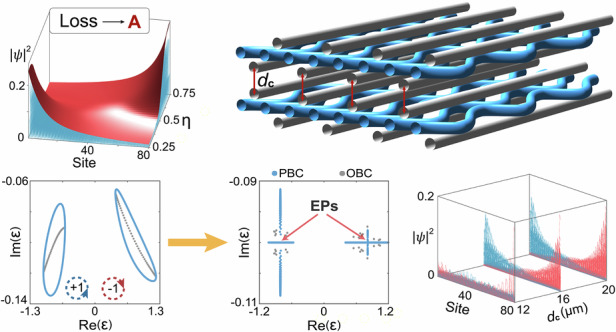

## Abstract (web summary)

We unveil that coupling two non-Hermitian systems with independent skin modes can induce tunable exceptional points, serving as a versatile system to reveal their interplay.

## Introduction

Non-Hermitian physics extends the principle of quantum mechanics to systems that no longer adhere to Hermiticity, offering a broader framework to describe nonequilibrium open systems^1–3^. The non-Hermitian formulation allows the Hamiltonian to have complex eigenvalues and nonorthogonal eigenstates, engendering unique and exotic phenomena that have no counterpart in Hermitian systems. Among them, the existence of exceptional points (EPs) has attracted significant interest, at which both the eigenvalues and eigenstates coalesce, serving as a phase transition point of non-Hermitian systems^4–6^. The EP possesses a self-intersecting energy topology, which exhibits fruitful physical consequences that can be employed for various applications^7–18^. Another intriguing hallmark of non-Hermitian systems is the non-Hermitian skin effect (NHSE)^19–27^. In this case, a multitude of eigenstates are localized at the boundary as skin modes, accompanied by the intense sensitivity of the bulk states to the boundary conditions. Such feature leads to the breakdown of traditional bulk-boundary correspondence, prompting a reexamination of the impact of non-Hermicity on topological phases^28–32^. Correspondingly, the non-Bloch band theory has been developed to describe the feature of the skin modes^33,34^, accompanied by the discovery of many intriguing effects, such as higher-order skin effect^35–38^, hybrid skin-topological effect^39–43^ and dynamic skin effect^44–47^. Recently, investigating the connection between EPs and NHSE has garnered significant attention, with many efforts dedicated to elucidating their interplay^3,28,37,48–50^. However, when focusing on the framework of interaction within multiple non-Hermitian systems, the interplay between these two non-Hermitian hallmarks remains a considerably unexplored territory both theoretically and experimentally.

Here, we investigate the synergistic effects by coupling two non-Hermitian systems with independent NHSE and propose the concept of coupled NHSE, in which EPs can emerge and tune the coupled NHSE, thus revealing the interplay between EP and NHSE. Initially, we realize the skin effect in a dissipative photonic lattice with curved waveguides, where the skin effect arises from the interplay between the artificial gauge field (AGF) and on-site losses. We discover that the AGF, as well as the distribution of non-Hermitian losses, can serve as versatile degrees of freedom to manipulate the localization direction of the skin modes. Subsequently, we explore the coupled NHSE by coupling two subsystems with skin modes induced by different loss distributions. We find that multiple pairs of EPs appear in the momentum-space band structure, resulting in the deactivation of most skin modes and attenuation of the coupled NHSE. Once the loss distribution mismatches, however, the EPs disappear, thus alleviating the attenuation of the coupled NHSE. We experimentally demonstrate the concept of the interplay between EP and coupled NHSE by measuring the light propagation dynamics in photonic waveguide arrays fabricated by femtosecond laser direct writing techniques.

## Results

### Effective coupling induced by AGF

**Fig. 1:**
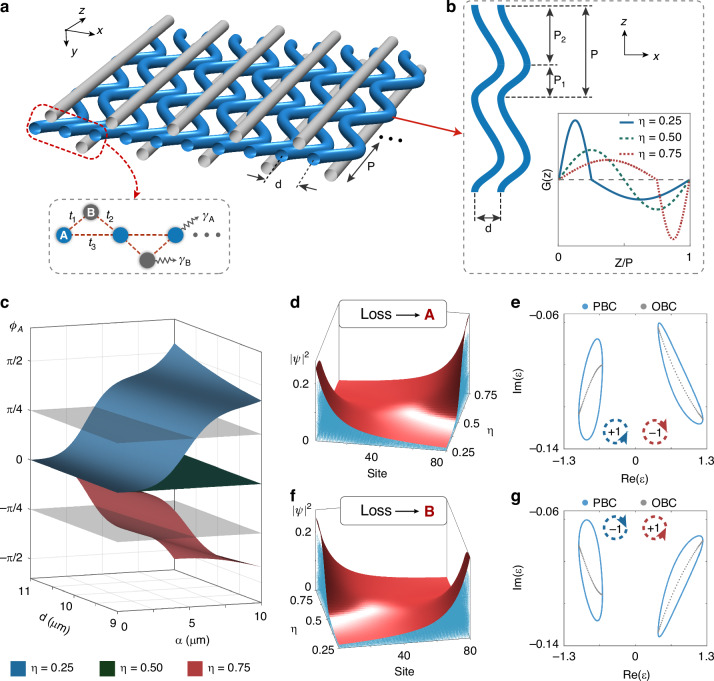
**Non-Hermitian photonic waveguides with skin effects.** **a** Schematic diagram of a non-Hermitian photonic system consisting of curved waveguides A and straight waveguides B. **b** Trajectory of the curved waveguides. The inset represents the function of AGF for different $\eta$. **c** The phase of ${\kappa }_{{eff}}$ with respect to the change of bending amplitude $a$ and coupling distance $d$ corresponding to different $\eta$. **d**, **f** The eigenfunction distributions of the system with respect to the change of $\eta$, where non-Hermitian losses are introduced into the sublattices A (**d**) and B (**f**). **e**, **g** The quasienergy spectra of the system when the on-site losses are introduced into the sublattices A (**e**) and B (**g**)

We start with a one-dimensional photonic waveguide lattice as illustrated in Fig. 1a. The lattice is composed of sublattices A and B with on-site losses denoted by ${\gamma }_{A}$ and ${\gamma }_{B}$, respectively. The sublattice A is arranged in the middle layer with equal spacing sites, while the sublattice B is alternately arranged in the upper and lower layers. The unique arrangement introduces nearest-neighbor couplings ${t}_{1}$ and ${t}_{2}$ as well as a next-nearest-neighbor coupling ${t}_{3}$ acting between sublattices A, where the complexity of ${t}_{3}$ is essential for achieving the NHSE (see detailed explanation in Supplementary Note 1). Here, we periodically modulate the waveguides A in a piecewise bending trajectory $x(z)$ along the propagation direction, with detailed parameters illustrated in Fig. 1b. In each modulation period *P*, we apply two kinds of sinusoidal modulations with the same amplitude $a$ but different periods ${P}_{1}$ and ${P}_{2}$, wherein we define $\eta ={P}_{1}/P$. Under the condition of paraxial approximation, the Hamiltonian of the non-Hermitian system can be described as

1 $$H\left(z\right)=\mathop{\sum }\limits_{i=1}^{N}[({\beta }_{A}-i{\gamma }_{A}){a}_{i}^{\dagger }{a}_{i}+{(\beta }_{B}-i{\gamma }_{B}){b}_{i}^{\dagger }{b}_{i}]+\mathop{\sum }\limits_{i=1}^{N}{t}_{1}\left(z\right){a}_{i}^{\dagger }{b}_{i}+\mathop{\sum }\limits_{i=1}^{N-1}{t}_{2}\left(z\right){b}_{i}^{\dagger }{a}_{i+1}+\mathop{\sum }\limits_{i=1}^{N-1}{t}_{3}\left(z\right){a}_{i}^{\dagger }{a}_{i+1}+H.C.$$

where *N* is the number of waveguides A (B), ${a}_{i}^{\dagger }$ (${b}_{i}^{\dagger }$) and ${a}_{i}$ (${b}_{i}$) are the creation and annihilation operators of waveguides A (B). The propagation constants of the curved waveguide A and straight waveguide B are donated by ${\beta }_{A}$ and ${\beta }_{B}$, respectively. Driven by the periodic bending modulation, an additional phase is introduced via the Peierls substitution^51–53^, resulting in ${t}_{3}\left(z\right)={\kappa }_{0}{e}^{i{\boldsymbol{G}}\left(z\right){{\boldsymbol{r}}}_{{mn}}}$, where ${\boldsymbol{G}}\left(z\right)={k}_{0}\partial x/\partial z{{\boldsymbol{e}}}_{{\boldsymbol{x}}}$ is the AGF induced by bending trajectory (see the inset in Fig. 1b), ${k}_{0}=2\pi {n}_{0}/\lambda$ is the wave number in the background medium, ${\kappa }_{0}$ is the coupling strength without bending and ${{\boldsymbol{r}}}_{{mn}}$ is the corresponding distance pointing from site *m* to *n*. In the high-frequency limit ($2\pi /P\gg {\kappa }_{0}$), we obtain an effective Hamiltonian ${H}_{{eff}}$ (see detailed calculation in Supplementary Note 2) from our modulated system and ${t}_{3}$ can be equivalent to an effective coupling ${\kappa }_{{eff}}=\frac{{\kappa }_{0}}{P}{\int }_{0}^{P}{e}^{i{\boldsymbol{G}}\left(z\right){{\boldsymbol{r}}}_{{mn}}}{dz}=|{\kappa }_{{eff}}|{e}^{i{\phi }_{A}}$. To explore the modulation of AGF, we calculate the phase term ${\phi }_{A}$ with respect to the change of bending amplitude $a$ and coupling distance $d$ in Fig. 1c. We find that ${\phi }_{A}$ possesses high stability with the change of $d$. As $a$ increases, ${\phi }_{A}$ remains zero at $\eta =0.5$, while gradually increases (decreases) when $\eta < 0.5$ ($\eta > 0.5$) so that ${\kappa }_{{eff}}$ becomes a complex number.

### Tunable NHSE

We first introduce losses solely to the waveguides A and calculate their eigenstates under open boundary conditions (OBC) from the effective Hamiltonian ${H}_{{eff}}$ in Fig. 1d, where the bulk eigenstates are found to be localized at the left and right boundary when $\eta < 0.5$ and $\eta > 0.5$, respectively, reflecting the existence of skin modes. In contrast, the skin effect disappears when $\eta =0.5$. Figure 1e shows the complex quasienergy spectrum under periodic boundary conditions (PBC) and OBC. We can see that the quasienergy spectrum exhibits two discrete closed loops under PBC, while it collapses into two discrete arcs under OBC. It is noteworthy that the quasienergy trajectory under OBC is enclosed by that under PBC, where the winding direction under PBC is related to the localization direction of the eigenstates under OBC. This can be characterized by a winding number $w=\frac{1}{2\pi i}{\int }_{0}^{2\pi }{\partial }_{k}\log \det \left[{H}_{{eff}}\left(k\right)-{E}_{0}\right]{dk}$^19,54,55^, where ${E}_{0}$ is any given reference quasienergy. By setting $\eta < 0.5$($\eta > 0.5$), the quasienergy spectrum under OBC is enclosed counterclockwise (clockwise) by that under PBC. The corresponding winding number $w=1$ ($w=-1$) reveals the direction of NHSE, i.e., the eigenstates are localized at the left (right) boundary. Interestingly, we find that the localization direction of skin modes is reversed when the waveguides B are endowed with on-site losses, which can be obviously reflected through the distribution of all eigenstates in Fig. 1f. Figure 1g plots the complex quasienergy spectrum and the corresponding winding numbers $w=-1$ and $w=1$ are associated with the case of $\eta < 0.5$ and $\eta > 0.5$, respectively.

**Fig. 2:**
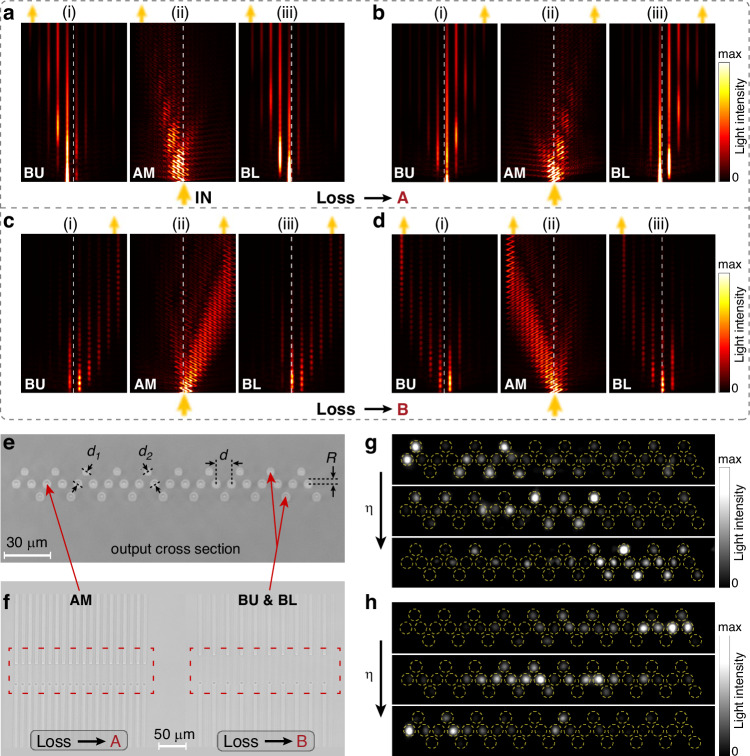
**Numerical and experimental results of the NHSE.** **a**, **b** Numerical simulations of light intensity distributions in the photonic system with $\eta =0.25$ (**a**) and 0.75 (**b**), where non-Hermitian losses are introduced into waveguides A with ${\gamma }_{A}=0.2{\rm{m}}{{\rm{m}}}^{-1}$. In each figure, the three insets from left to right represent the simulated light intensity distributions in the upper layer (marked as BU), middle layer (marked as AM) and lower layer (marked as BL), respectively. **c**, **d** Numerical simulations for $\eta =0.3$ (**c**) and 0.7 (**d**), where non-Hermitian losses are introduced into waveguides B with ${\gamma }_{B}=0.2{\rm{m}}{{\rm{m}}}^{-1}$. **e**, **f** The microscopic photograph of fabricated samples (**e**) and breaking lines (**f**). **g**, **h** Experimentally measured light intensity distributions at the output facet of the sample with $\eta =0.25$, $0.5$ and 0.75 for ${\gamma }_{A}=0.2{\rm{m}}{{\rm{m}}}^{-1}$ (**g**) and $\eta =0.3$, $0.5$ and 0.7 for ${\gamma }_{B}=0.2{\rm{m}}{{\rm{m}}}^{-1}$ (**h**). The structure parameters are $P=2{\rm{mm}}$, ${d}_{1}=9.5{\rm{\mu }}{\rm{m}}$, ${d}_{2}=9{\rm{\mu }}{\rm{m}}$, $d=10{\rm{\mu }}{\rm{m}}$, $a=5.5{\rm{\mu }}{\rm{m}}$ and $R=3{\rm{\mu }}{\rm{m}}$

To confirm the theoretical analysis, we simulate the propagation of light with a wavelength of 808 nm in the coupled waveguide arrays using the commercial software RSoft. We first introduce losses into waveguides A. The waveguide radius and refractive index contrast between the waveguide and background are $3{\rm{\mu }}{\rm{m}}$ and 0.004, respectively. The initial excitation is launched into the center of the waveguide array. When $\eta =0.25$, as shown in Fig. 2a, light is found to propagate towards the left boundary. On the contrary, the propagation direction of light is reversed and points to the right boundary at $\eta =0.75$, as shown in Fig. 2b. Such phenomenon is since that ${\phi }_{A}$ with $\eta =0.75$ is opposite to that with $\eta =0.25$, which results in the different localization directions. Similarly, we show the numerical results when on-site losses are introduced into waveguides B. In this case, the localization direction of the skin modes is reversed. The corresponding numerical results with $\eta =0.3$ and $\eta =0.7$ are given in Fig. 2c, d, where the propagation of an injected light at the center points to the right and left boundary, respectively, as expected.

In the experiment, we fabricated the photonic waveguide array inside boroaluminosilicate glass by employing the femtosecond laser direct writing technique (see “Materials and methods” and Supplementary Note 5 for experimental details). Figure 2e shows the microscopic photograph of the fabricated sample at the output facet. The non-Hermitian losses in waveguides are introduced by setting breaking lines periodically inside the waveguide (Fig. 2f). We inject a laser beam with a wavelength of 808 nm at the center of the array. In the case where on-site losses are introduced in waveguides A, the experimentally measured light intensity distributions at the output facet are given in Fig. 2g. We observe that light is mainly concentrated on the left boundary of the waveguide array with $\eta =0.25$ (upper inset), stays at the center with $\eta =0.5$ (middle inset), while gathers at the right boundary with $\eta =0.75$ (lower inset). In contrast, the results with on-site losses introduced in waveguides B indicate an opposite trend with increasing $\eta$ (Fig. 2h). These experimental results successfully demonstrate the feature of the NHSE and the topological transition of the non-Hermitian skin modes, indicating that AGF serve as a powerful tool for tuning the localization direction of the skin modes.

### EPs in a coupled system with coupled NHSE

**Fig. 3:**
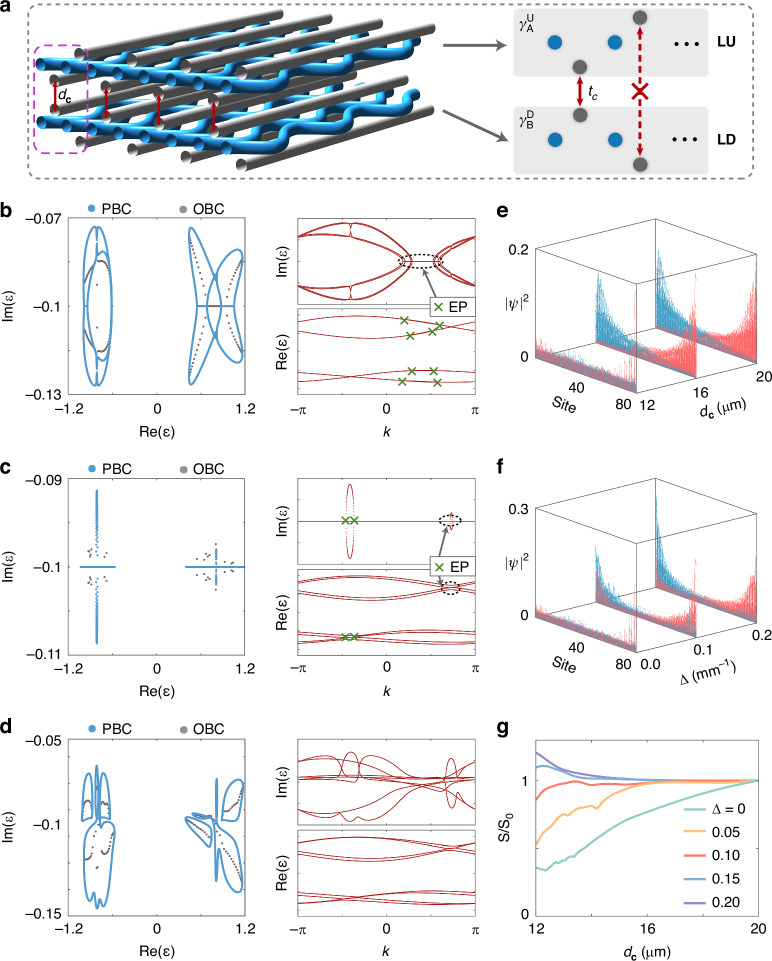
**EPs in a coupled system with skin modes.** **a** Schematic diagram of a coupled system with two non-Hermitian chains, labeled as LU and LD. **b**–**d** Complex quasienergy spectra of the coupled system with ${d}_{c}=16{\rm{\mu }}{\rm{m}}$ and $\Delta =0$ (**b**), ${d}_{c}=12{\rm{\mu }}{\rm{m}}$ and $\Delta =0$ (**c**), ${d}_{c}=12{\rm{\mu }}{\rm{m}}$ and $\Delta =0.1{\rm{m}}{{\rm{m}}}^{-1}$ (**d**). **e**, **f** The eigenfunction distributions in LU (blue) and LD (red) with respect to the change of ${d}_{c}$ (**e**) and $\Delta$ (**f**). **g** The calculated skin factor with respect to the change of ${d}_{c}$ and $\Delta$, where ${S}_{0}$ is the skin factor with ${d}_{c}=20{\rm{\mu }}{\rm{m}}$

After realizing the NHSE, we investigate the coupled NHSE, i.e., coupling two systems with independent NHSE and revealing the interplay between loss-induced skin modes and EPs. We apply the same bending modulation to two spatially completely symmetric photonic waveguide arrays (labeled as LU and LD, respectively) and couple them via waveguides B with coupling ${t}_{c}$, which is illustrated in Fig. 3a. Note that each unit cell contains eight waveguides, where only the nearest pair of waveguides B is coupled. We introduce losses to waveguides A in LU and waveguides B in LD, and define $\Delta ={\gamma }_{A}^{U}-{\gamma }_{B}^{D}$ as the loss difference. The corresponding Bloch Hamiltonian under PBC takes the form

2 $${H}_{c}(k)=\left[\begin{array}{cc}{H}_{U}(k) & {H}_{T}\\ {H}_{T} & {H}_{D}(k)\end{array}\right]$$

where the subsystem LU/LD is described as

3 $${H}_{U/D}\left(k\right)=\left[\begin{array}{cc}1 & 0\\ 0 & 1\end{array}\right]\otimes \left[\begin{array}{cc}{\beta }_{A}-i{\gamma }_{A}^{U/D} & {t}_{1}\\ {t}_{1} & {\beta }_{B}-i{\gamma }_{B}^{U/D}\end{array}\right]+\left[\begin{array}{cc}0 & 1\\ {e}^{{ik}} & 0\end{array}\right]\otimes \left[\begin{array}{cc}{t}_{3}^{* }{e}^{-{ik}}+{t}_{3} & {t}_{2}{e}^{-{ik}}\\ {t}_{2} & 0\end{array}\right]$$

${H}_{T}$=$$\left[\begin{array}{cc}1 & 0\\ 0 & 0\end{array}\right]\otimes \left[\begin{array}{cc}0 & 0\\ 0 & {t}_{c}\end{array}\right]$$ represent the coupling between the subsystems. We first add equal losses ($\Delta =0$), which results in skin modes localized toward the left boundary in LU and right boundary in LD, respectively. When ${t}_{c}=0$, the corresponding quasienergy spectrum under PBC exhibits the superposition of that in Fig. 1e, g. Specifically, the eight bands can be divided into four pairs, and for each two-band pair, the real parts completely coincide and the imaginary parts intersect at a critical point $k={k}_{c}$ with $k$ being the Bloch momentum (see Fig. S11). We can treat each two-band pair as a two-level system with an effective momentum-space Hamiltonian

4 $${H}^{{\prime} }(k)=\left[\begin{array}{cc}{\beta }_{0}\left(k\right)-i{\gamma }_{1}\left(k\right) & {t}_{c}\\ {t}_{c} & {\beta }_{0}\left(k\right)-i{\gamma }_{2}\left(k\right)\end{array}\right]$$

where ${\gamma }_{1}\left({k}_{c}\right)={\gamma }_{2}\left({k}_{c}\right)$. Especially, we emphasize that the relation ${\gamma }_{1}\left(k\right)+{\gamma }_{2}\left(k\right)={\gamma }_{A}^{U}$ is hold in the case of adding equal losses ($\Delta =0$). This indicates an underlying parity-time (PT) symmetry within our dual-lattice system, of which the detailed explanation is shown in Supplementary Note 7. Once ${t}_{c}\,\ne \,0$, the eigenvalues are $E\left(k\right)={\beta }_{0}\left(k\right)-i[{\gamma }_{1}\left(k\right)+{\gamma }_{2}\left(k\right)]/2\pm \sqrt{{t}_{c}^{2}-{[{\gamma }_{1}\left(k\right)-{\gamma }_{2}\left(k\right)]}^{2}/4}$, and one pair of EPs appear under the condition of |${\gamma }_{1}\left(k\right)-{\gamma }_{2}\left(k\right)$|$=2{t}_{c}$. In this regard, the EP pair is spawned from the critical point at $k={k}_{c}$ and the length of the exact phase (i.e., the distance between the two EPs in momentum space) would get enlarged by increasing ${t}_{c}$. To demonstrate this point, we choose a relatively small ${t}_{c}$ and show the calculated quasienergy spectrum in Fig. 3b. We find that the spectrum under PBC begins to collapse due to the appearance of four pairs of EPs, such that the spectrum under OBC is partially enclosed. As ${t}_{c}$ further increases, the length of exact phase almost tends to $2\pi$, thereby causing the quasienergy spectrum under PBC to collapse into arcs, with the corresponding area shrinking significantly. (Fig. 3c).

### Breakdown of coupled NHSE induced by EPs

The EPs induced spectrum change could strongly affect the coupled skin modes. We calculate the eigenfunction distributions for different coupling gap distance ${d}_{c}$ in Fig. 3e, where the blue and red curves are associated with the eigenfunctions in LU and LD, respectively. We note that the coupled NHSE is significantly weakened as ${d}_{c}$ decreases, since the quasienergy spectrum under OBC can no longer be well enclosed by that under PBC with an EP-induced shrinked area, leading to the consequence that the eigenmodes of the system no longer exhibit the skin effect. Moreover, we add that the above phenomenon is also directly manifested in the quasienergy spectrum under OBC. Here, the emergence of exceptional points (EPs) drives a phase transition from skin modes to extended bulk modes, thereby resulting in the delocalization of the system (see Supplementary Note 6 for discussions).

When we introduce unequal losses in the waveguide arrays, however, the EPs are absent as shown in Fig. 3d (also see Supplementary Note 6 for detailed explanation). As a result, the quasienergy spectrum under PBC still keeps a finite area, instead of collapsing into arcs, which maintains the existence of massive skin modes. Figure 3f plots the calculated eigenfunction distributions for different $\Delta$ with ${d}_{c}$ being fixed at $12{\rm{\mu }}{\rm{m}}$, where we can observe the significant localization feature at $\Delta \ne 0$. Especially, the localization feature is improved as $\Delta$ increases. In order to quantify the localization feature of eigenstates in our proposed coupled non-Hermitian system directly, we define a skin factor $S={{\mathop{\sum}\nolimits_{j=1}^{N/2}}}[\mathop{\sum }\nolimits_{i=1}^{N}({{\left|{\psi }_{i,j}^{U}\right|}^{2}+\left|{\psi }_{i,j}^{D}\right|}^{2}){e}^{\left|j-N/4\right|}]$ to fundamentally constitutes a weighted summation of probability densities across all eigenstates at spatial lattice points, where ${\left|{\psi }_{i,j}^{U\left(D\right)}\right|}^{2}$ denotes the probability density of the $i$-th eigenstate at lattice site $j$ corresponding to LU (LD). The weighting function ${e}^{\left|j-N/4\right|}$ grows exponentially with boundary proximity, significantly amplifying contributions from boundary-proximal eigenstates. Thus, by definition, a higher skin factor indicates a better skin effect. Figure 3g shows the calculated skin factor as a function of ${d}_{c}$ for different $\Delta$. The phenomenon of EPs accelerated breakdown of coupled NHSE is evident, i.e., the skin factor with $\Delta =0$ drops dramatically by decreasing ${d}_{c}$, while in contrast, it maintains a higher value when $\Delta \ne 0$.

**Fig. 4:**
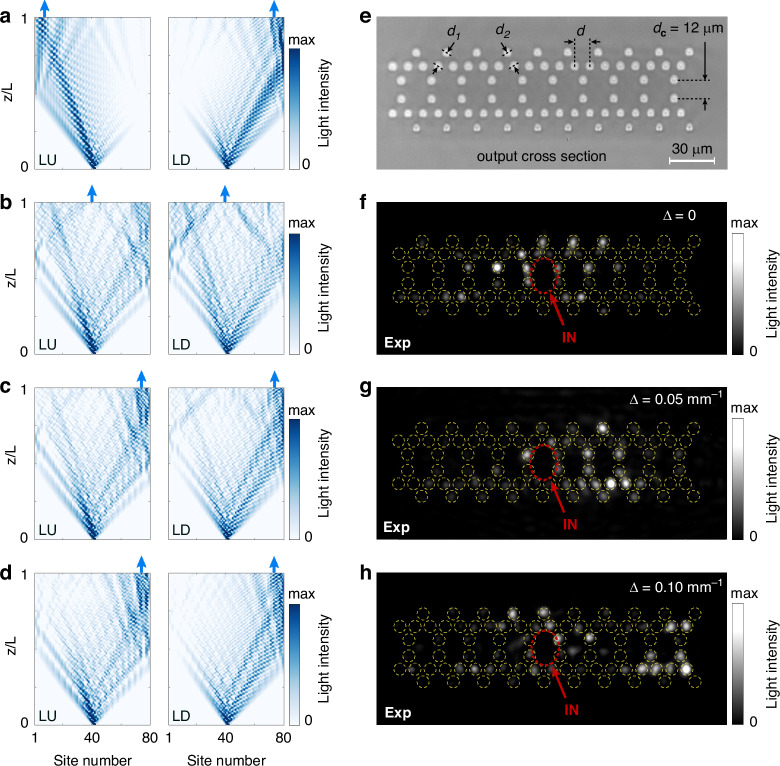
**EPs induced the breakdown of the coupled NHSE.** **a**–**d** Numerically calculated evolution of light in the coupled waveguide arrays with ${t}_{c}=0$ and $\Delta =0$ (**a**), ${d}_{c}=12{\rm{\mu }}{\rm{m}}$ and $\Delta =0$ (**b**), ${d}_{c}=12{\rm{\mu }}{\rm{m}}$ and $\Delta =0.05{\rm{m}}{{\rm{m}}}^{-1}$ (**c**), ${d}_{c}=12{\rm{\mu }}{\rm{m}}$ and $\Delta =0.1{\rm{m}}{{\rm{m}}}^{-1}$ (**d**). The results are renormalized at each propagation distance *z*. **e** The microscopic photograph of fabricated samples at the output facet. The corresponding structure parameters are $P=2{\rm{mm}}$, $a=5.5{\rm{\mu }}{\rm{m}}$, ${d}_{1}=9.5{\rm{\mu }}{\rm{m}}$, ${d}_{2}=9{\rm{\mu }}{\rm{m}}$ and $d=10{\rm{\mu }}{\rm{m}}$. **f**–**h** The experimentally measured light intensity distributions at the output facet of the sample corresponding to the cases in (**b**), (**c**) and (**d**), respectively

To prove the theoretical analysis, we numerically calculate the light evolution in the coupled system. The waveguide arrays LU and LD are excited simultaneously at the center region. We first consider the case with $\Delta =0$. Figure 4a shows the results with ${t}_{c}=0$, where the light waves in LU and LD propagate independently and exhibit a trend of left localization and right localization, respectively. When ${d}_{c}=12{\rm{\mu }}{\rm{m}}$, the coupled NHSE is overcome to a great extent due to the existence of multiple EPs, which cause the collapse of the quasienergy spectrum under PBC (Fig. 4b). When we set $\Delta \ne 0$, however, the coupled NHSE recovers in Fig. 4c, d since the EPs are absent. One should note that since the waveguide array LD possesses a lower loss, the localization trend in LD dominates the wave transmission, such that light waves in LU and LD are both localized on the right boundary. In addition, the localization feature in Fig. 4d can also be observed to be stronger than that in Fig. 4c due to a larger $\Delta$.

In the experiment, we fabricated three samples with $\Delta =0$, $\Delta =0.05{\rm{m}}{{\rm{m}}}^{-1}$ and $0.1{\rm{m}}{{\rm{m}}}^{-1}$, respectively, where ${d}_{c}$ is chosen as $12{\rm{\mu }}{\rm{m}}$ (see experimental details in Supplementary Note 5). The corresponding microscopic photograph of the fabricated sample at the output facet is shown in Fig. 4e. By measuring output light diffraction patterns, we can observe that light is disorderly distributed at $\Delta =0$ as shown in Fig. 4f. Once the loss difference is introduced, Fig. 4g, h show that light gradually gathers to the right boundary during propagation because of the disappearance of EPs, which is consistent with our theory.

## Conclusions

In summary, we have demonstrated the NHSE in photonic waveguide arrays with AGF, where both the AGF and non-Hermitian loss can be used to tune the localization direction of the skin modes. We have studied a coupled system with two waveguide arrays possessing skin modes localized in opposite directions. We discovered that the coupled system supports multiple pairs of EPs, which can result in the shrinking of the quasienergy spectrum under PBC and, therefore, the breakdown of coupled NHSE. In contrast, the coupled NHSE could be recovered when EPs are absent. Our work has revealed the interplay between the two hallmarks of non-Hermitian systems, which may find applications for manipulating non-Hermitian skin modes using EPs and open avenues for non-Hermitian applications in topological matter, wave transport, and quantum sensors.

## Materials and methods

### Sample fabrication and measurement

The fabrication of photonic waveguides employed the technique of femtosecond laser direct writing. In the experiment, we exploited a Ti:sapphire laser (Light Conversion Carbide 5 W) to focus inside the boroaluminosilicate glass. To precisely control the movement speed of the glass, we utilized an Aerotech micro-displacement platform. This meticulous operation induced a slight change of refractive index (~0.004) in the focal region, successfully creating a single-mode parabolic photonic waveguide with a circular cross-section of about $6{\rm{\mu }}{\rm{m}}$ in diameter. By finely adjusting the writing speed, we can fine-tune the effective modal index of the waveguide, achieving modulation of the propagation constant in the non-Hermitian Hamiltonian (see Fig. S8a). Furthermore, the coupling strength could be controlled by meticulously selecting the appropriate gap between adjacent waveguides, which is another key parameter in the non-Hermitian Hamiltonian (see Fig. S8b).

### Technique to introduce non-Hermitian losses

The on-site losses of the waveguide mode are effectively introduced through the application of a breaking line technique. Specifically, in the process of laser direct writing, the laser is periodically switched so that the waveguide is uniformly broken along the propagation direction. By controlling the turn-off time of the laser, the waveguide can be endowed with an adjustable loss. Figure S9a shows the relationship between the loss coefficient $\gamma$ and breaking length. We can see that the change of $\gamma$ is more sensitive to the large breaking length, such that a smaller break length is selected to guarantee the accuracy of the loss in the experiment. To introduce a substantial loss in the waveguide, we adopt a method involving the insertion of multiple breaking lines within each period. In particular, we note that $\gamma$ satisfies a linear relationship with respect to the breaking number (see Fig. S9b), which ensures the accuracy of the loss to a great extent.

## Supplementary information

Supplementary Information for Coupled non-Hermitian skin effect with exceptional points

## Data Availability

The data that support the findings of this work are available from the corresponding authors upon reasonable request.
