# Supplementary material for: Coupled non-Hermitian skin effect with exceptional points
Source: Light Sci Appl. 2025 Sep 23;14:339. doi: 10.1038/s41377-025-02006-6 (PMC12457596; doi:10.1038/s41377-025-02006-6)
Supplement: Supplementary file 1 — Supplementary Information for Coupled non-Hermitian skin effect with exceptional points [file 41377_2025_2006_MOESM1_ESM.pdf]

Supplementary Information for
Coupled non-Hermitian skin effect with exceptional points

Guo-Huai Wang[1], Ran Tao[1], Zhen-Nan Tian[1], Qi-Dai Chen[1], and Xu-Lin Zhang[1]

[1]State Key Laboratory of Integrated Optoelectronics, JLU Region, College of Electronic Science and Engineering, Jilin University, Changchun 130012, China

Correspondence: Xu-Lin Zhang (xulin_zhang@jlu.edu.cn) or Qi-Dai Chen (chenqd@jlu.edu.cn) or Zhen-Nan Tian (zhennan_tian@jlu.edu.cn).

These authors contributed equally: Guo-Huai Wang, Ran Tao.

This Supplementary Information contains 7 Supplementary Notes and 14 Figures.

Supplementary Note 1: A theoretical model for realizing the NHSE.

Non-Hermitian skin effect (NHSE) has been successfully observed in various systems, such as acoustic systems[1,2], electrical circuits[3,4], quantum systems[5] and mechanics systems[6]. These systems are nonreciprocal systems where nonreciprocal hopping is required to achieve the skin modes. The unique localization feature makes this non-Hermitian effect especially useful for manipulating light for photonic applications. However, optical systems are typically reciprocal systems, which are difficult to support non-Hermitian skin modes. Recently, it was proposed that non-Hermitian skin modes can be alternatively realized in reciprocal systems whereas non-Hermitian gain/loss and reciprocal but complex hopping are needed[7-11]. Here we propose a one-dimensional dissipative photonic lattice as illustrated in Fig. S1 to realize the NHSE.

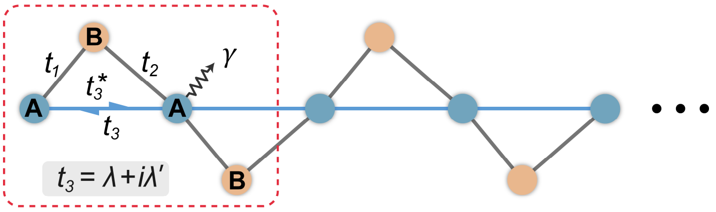

Fig. S1 Schematic diagram of a one-dimensional photonic lattice. The sublattices A are arranged in the middle layer and the sublattices B are alternately arranged in the upper and lower layers.

Under the periodic boundary condition (PBC), the corresponding Bloch Hamiltonian is written as

$$H(k) = \begin{bmatrix} t_3^* e^{-ik} + t_3 e^{ik} - i\gamma & t_1 + t_2 e^{-ik} \\ t_1 + t_2 e^{ik} & 0 \end{bmatrix} \tag{S1}$$

where k is the Bloch momentum, t_1 and t_2 are real-valued nearest-neighbor couplings, while $t_3 = \lambda + i\lambda'$ is complex-valued next-nearest-neighbor coupling acting between the sublattices A. The on-site losses are denoted by γ. We show the eigenfunctions under open boundary condition (OBC) in Fig. S2a-c and find that an extensive number of eigenstates are localized at the left and right boundary when $\lambda' > 0$ and $\lambda' < 0$, respectively, exhibiting the feature of the skin modes. In contrast, the eigenstates are no longer localized at the boundary when $\lambda' = 0$. Such phenomena can be manifested in energy spectra under PBC and OBC as shown in Fig. S2d-f. We can see that the energy spectrum exhibits two discrete closed loops when $\lambda' \neq 0$, while collapses to arcs when $\lambda' = 0$, of which the topology can be characterized by a winding number[12,13]

$$w = \frac{1}{2\pi i}\int_0^{2\pi} \partial_k \log\det\left[H(k) - E_0\right] dk \tag{S2}$$

where E_0 is any given reference energy.

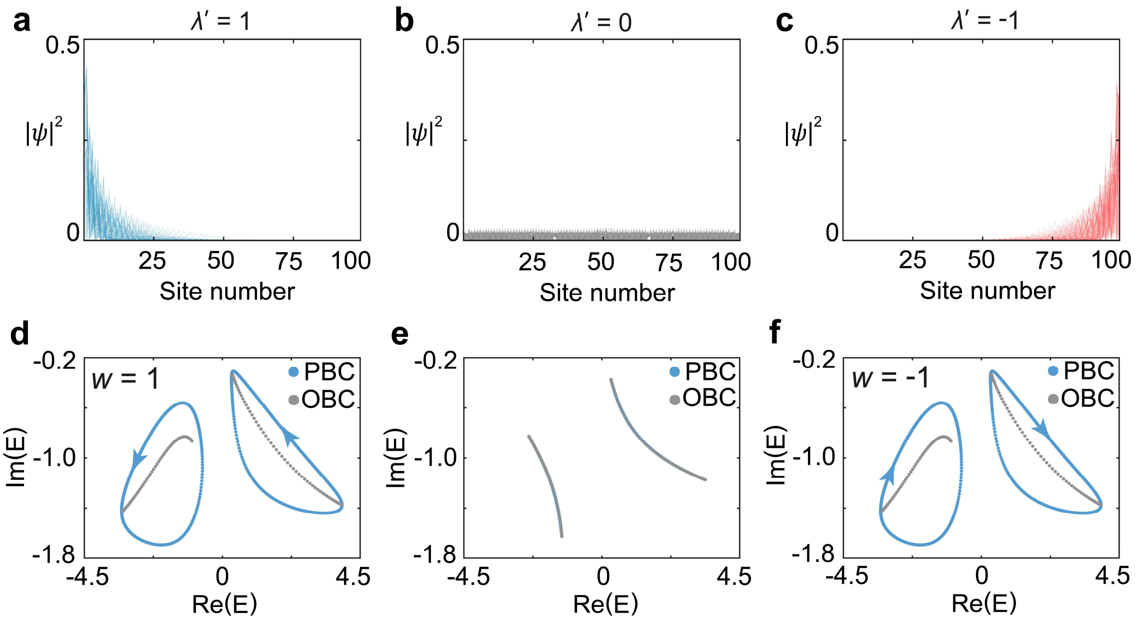

Fig. S2 **a-c** The eigenfunctions of the photonic lattice with $\lambda' = 1$ (**a**), $\lambda' = 0$ (**b**) and $\lambda' = -1$ (**c**). **d-f** The corresponding complex energy spectra under PBC (blue dots) and OBC (gray dots). At $\lambda' = 1$ ($\lambda' = -1$), the spectrum forms two closed loops and possesses a winding number of $w = 1$ ($w = -1$). The other parameters are $t_1 = 2$, $t_2 = 1$, $\lambda = 1/2$ and $\gamma = 2$.

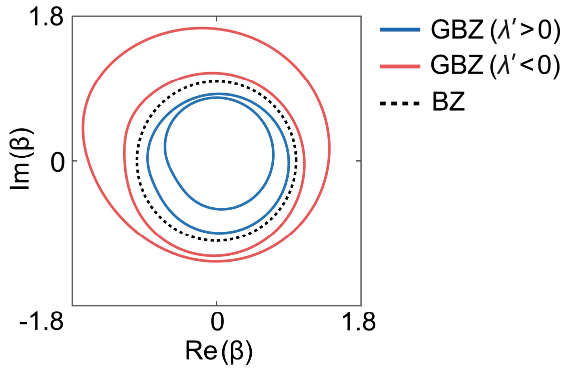

Fig. S3 The GBZs corresponding to $\lambda' > 0$ (blue circle) and $\lambda' < 0$ (red circle).

We further demonstrate the results using generalized Brillouin zone (GBZ) in Fig. S3. The black dotted line represents the unit cycle, corresponding to the Brillouin zone (BZ). In the case of on-site losses in waveguides A, the GBZ distributes in the inside of the unit circle and separates into two closed loops when $\lambda' > 0$, while distributes in the outside of the unit circle when $\lambda' < 0$, indicating eigenstates are localized in the left and right boundary, respectively. In the main text, we successfully

realize a complex-valued next-nearest-neighbor coupling by introducing artificial gauge fields (AGFs), and experimentally confirm the feasibility of the above model.

Supplementary Note 2: Effective Hamiltonian of the periodically modulated system

In Floquet systems, the Hamiltonian exhibits temporal periodicity, i.e., $H(z) = H(z+P)$ (here the propagation coordinate "z" acts as "time"). Thus, an effective Hamiltonian H_{eff} can be defined via the time-evolution operator over full period P, i.e., $U(P) = e^{-iH_{eff}P}$, hereby capturing the properties of the system's eigenstates at the end of each period ($z = nP$). Under the condition of high-frequency approximation ($2\pi/P \gg \kappa_0$, where κ_0 is the coupling strength between the waveguides), the system cannot adiabatically follow the rapid oscillations of the drive period. As a result, the Hamiltonian $H(z)$ can be approximated as the effective Hamiltonian H_{eff} during the whole evolution process, which then describes the average behavior of the system throughout the drive period. In the following, we give detailed discussions on how an effective Hamiltonian can be obtained by using Floquet theory[14,15].

(1) ***Time-averaged effective Hamiltonian***. Since the system is modulated by periodic bending, the corresponding Hamiltonian $H(z)$ in the main text satisfies $H(z+P) = H(z)$, which is the embodiment of Floquet gauge modulation. Here we obtain the effective Hamiltonian $H_{eff} = \sum_{n=1}^{\infty} H_f^{(n)}$ by using Van Vleck expansion[16], and the first two orders are defined as

$$H_f^{(1)} = H_0 \tag{S3}$$

$$H_f^{(2)} = \sum_{m\neq 0} \frac{H_m H_{-m}}{m\omega} \tag{S4}$$

where

$$H_m = \frac{1}{P}\int_0^P e^{-im\omega z} H(z)\, dz \tag{S5}$$

corresponds to the Fourier transform of the Hamiltonian $H(z)$. To facilitate the subsequent discussion, we rewrite the above equation as

$$H_f^{(1)} = \frac{1}{P}\int_0^P H(z)\, dz \tag{S6}$$

and

$$H_f^{(2)} = \frac{-i}{2P}\int_O^P dz_1 \int_0^{z_1}\left(1-\frac{2(z_1-z_2)}{P}\right)[H(z_1),H(z_2)]\,dz_2 \tag{S7}$$

Here, $H_f^{(1)}$ is the leading order, equaling the average of the Hamiltonian over one period, while $H_f^{(2)}$ is considered as a correction term. It should be noted that we only consider the leading term of the effective Hamiltonian and ignore the influence of correction term. We emphasize that this approximation is reasonable because the calculated $H_f^{(2)}$ is always much smaller than $H_f^{(1)}$. Therefore, the time-averaged effective Hamiltonian can be written as

$$H_{eff} = \sum_{i=1}^{N}[(\beta_A - i\gamma_A)a_i^\dagger a_i + (\beta_B - i\gamma_B)b_i^\dagger b_i]$$

$$+\sum_{i=1}^{N} t_1' a_i^\dagger b_i + \sum_{i=1}^{N-1} t_2' b_i^\dagger a_{i+1} + \sum_{i=1}^{N-1} t_3' a_i^\dagger a_{i+1} + H.C. \tag{S8}$$

with $t_j' = \frac{1}{P}\int_0^P t_j(z)\,dz$, where j =1, 2 and 3.

(2) ***Numerical result of the effective Hamiltonian.*** To verify the rationality of the aforementioned approximation, here we have also provided a numerical calculation method for effective Hamiltonian. For the time-evolution operator $U(z)$, the Floquet states can be regarded as the eigenstates over one driving period P and satisfy the function

$$U(P)\psi(z) = e^{-i\varepsilon P}\psi(z) \tag{S9}$$

and

$$U(P) = \mathcal{T}e^{-i\int_0^P H(z)dz} \tag{S10}$$

where $\mathcal{T}$ denotes the time-ordering operator, ε is the quasienergy of the Floquet system. Due to the z-periodic Hamiltonian $H(z+P) = H(z)$, we can introduce an effective time-independent Hamiltonian H_{eff} to interpret the time-evolution operator as $U(P) = \mathcal{T}e^{-iH_{eff}P}$, in which the effective Hamiltonian H_{eff} is defined as

$$H_{eff} = \frac{i}{P}\log\big(U(P)\big) \tag{S11}$$

Combining Eq. S10 and S11, we can obtain the numerically calculated effective Hamiltonian H_{eff}.

Supplementary Note 3: The Floquet NHSE in real waveguide array

For our real waveguide array, the geometric design of waveguides has an additional effect when introducing AGF, i.e., introducing an unexpected next-next-nearest-neighbor coupling $t_4(z)$, which is previously ignored. Fig. S4a shows the structure diagram of the photonic waveguides, from which we can intuitively find that the strength of t_4 depends on the bending amplitude a. Fig. S4b shows the fitted coupling strength of t_4 with respect to the change of propagation distance z, corresponding to the structure parameters of initial distances $d_1 = 9.5$ μm, $d_2 = 9$ μm,$d = 10$ μm, bending amplitude $a = 5.5$ μm and the period $P = 2$ mm for the waveguide array, where d_1, d_2 and d are the waveguide distances associated with the coupling term t_1, t_2 and t_3 at the starting point, respectively. Driven by the periodic bending modulation, the spatial positions of curved waveguides and straight waveguides are staggered along the propagation direction, causing the coupling coefficient between them to change periodically. Note that t_4 takes the maximum value at the dotted line marked by star and the minimum value at the end, which corresponds to the peak point of the bending modulation wave to the trajectory.

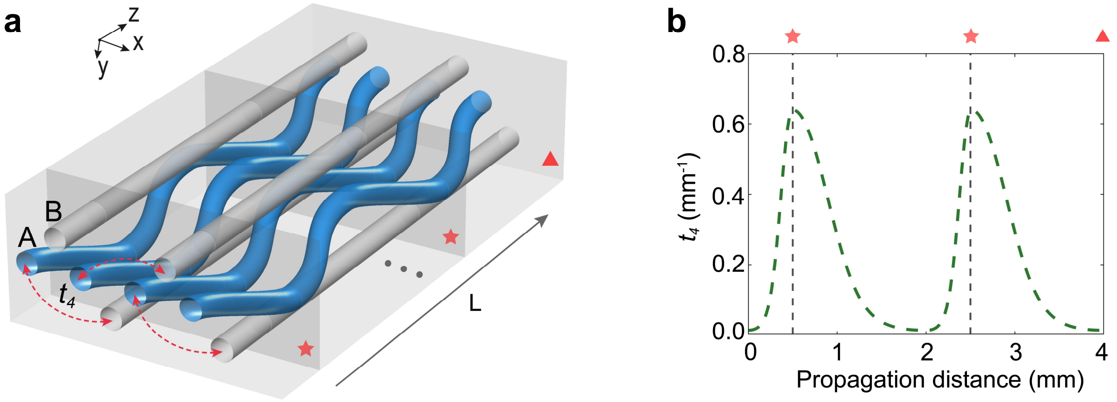

Fig. S4 a The structure diagram of the photonic waveguides with propagation length $L = 4$ mm, where a next-next-nearest-neighbor coupling t_4 is introduced (marked by red arrow). **b** The strength of t_4 with respect to the change of the propagation distance.

To further clarify the influence of t_4 on NHSE, we take the case where waveguides A are endowed with on-site losses of $\gamma_A = 0.2$ mm^{-1} as an example. We rewrite the effective Hamiltonian as

$$H_{eff} = \sum_{i=1}^{N}[(\beta_A - i\gamma_A)a_i^\dagger a_i + \beta_B b_i^\dagger b_i]$$

$$+\sum_{i=1}^{N} t_1' a_i^\dagger b_i + \sum_{i=1}^{N-1} t_2' b_i^\dagger a_{i+1} + \sum_{i=1}^{N-1} t_3' a_i^\dagger a_{i+1}$$

$$+\sum_{i=1}^{N-1} t_4' a_i^\dagger b_{i+1} + H.C. \tag{S12}$$

and calculate the eigenstates and eigenvalues. We can see that all eigenstates are localized at the left when $\eta = 0.25$ (see Fig. S5a), while at the right when $\eta = 0.75$ (see Fig. S5c), indicating the existence of the Floquet NHSE. The corresponding quasienergy spectra shown in Fig. S5d and S5f are similar with that shown in Fig. 1d in the main text and exhibit the same feature, corresponding to the winding number of $w = 1$ and $w = -1$, respectively. However, such feature is absent in eigenfunctions (see Fig. S5b) and quasienergy spectrum (see Fig. S5e) when setting $\eta = 0.5$, wherein the quasienergy spectrum under PBC collapses into lines drastically and coincides with that under OBC.

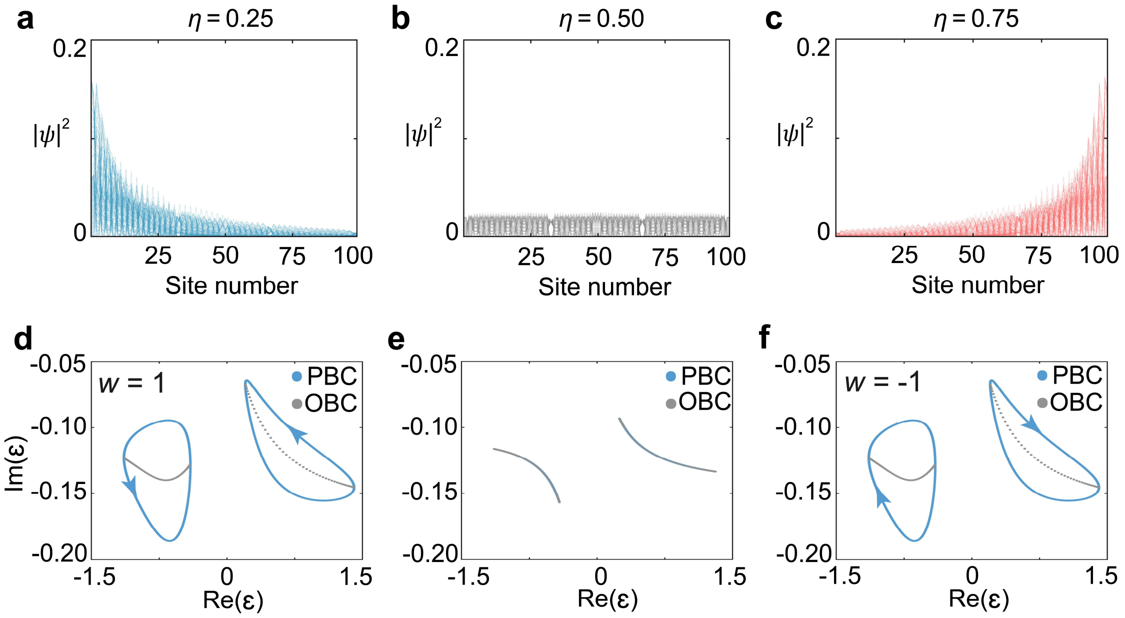

Fig. S5 a-c The eigenfunctions of the waveguide array with $\eta = 0.25$ (**a**), $\eta = 0.5$ (**b**) and $\eta = 0.75$ (**c**). **d-f** The corresponding complex quasienergy spectra under PBC (blue dots) and OBC (gray dots). At $\eta = 0.25$ ($\eta = 0.75$), the spectrum forms two closed loops and possesses a winding number of $w = 1$ ($w = -1$).

Moreover, we demonstrate the results of numerically calculated evolution of light so as to observe the localization direction of the skin modes intuitively. To start, we launch light from the waveguide located at the center of the waveguide array with site number $n = 100$. The waves gradually propagate to the left (right) and aggregate at the boundary as shown in Fig. S6a (Fig. S6c), corresponding to the localization direction of eigenfunctions, while exhibit discrete diffraction behavior at $\eta = 0.5$ as shown in Fig. S6b.

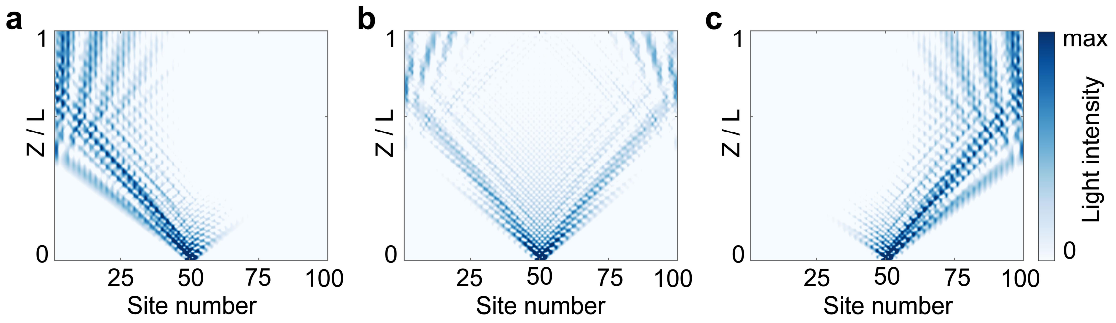

Fig. S6 Numerically calculated evolution of light for the case of $\eta = 0.25$ (**a**), $\eta = 0.5$ (**b**) and $\eta = 0.75$ (**c**), where waveguides A are endowed with on-site losses.

Since the above result is consistent with that in the main text, we emphasize that the interference of the extra coupling can be ignored.

Supplementary Note 4: Tuning the NHSE via on-site loss

We have mentioned in the main text that the distribution of the on-site losses within the waveguide array can tune the localization direction of the skin modes. We further add that the loss can serve as a degree of freedom to tune the localization strength of the skin modes.

For the waveguide array shown in Fig. 1a of the main text, we first consider the case where waveguides A are endowed with losses. As demonstrated by the eigenfunction distribution in Fig. S7a, we can see that the localization direction of skin modes always points toward the left boundary, while the localization strength exhibits significant attenuation with the decrease of loss. Such results clearly indicate that the loss can effectively tune the localization strength of the NHSE. On the other hand, the modulation of loss on the skin modes can also be reflected by corresponding quasienergy spectrum. In Fig. S7c, we show the quasienergy spectrum at $\gamma_A = 0.1\ \mathrm{mm}^{-1}$, where the separation degree between spectra under PBC and OBC is obviously reduced compared to that in Fig. 1e of the main text. In particular, the quasienergy spectrum overlaps completely when $\gamma_A = 0$, accompanied by the closure of the point gap, thereby resulting in the topological phase transition and delocalization of the eigenstates. Similarly, introducing losses exclusively to waveguides B yields similar phenomena regarding the modulation of localization strength. The difference is that the distribution of the losses changes the localization direction of the NHSE, which we have given the experimental verification in Fig. 2 of the main text.

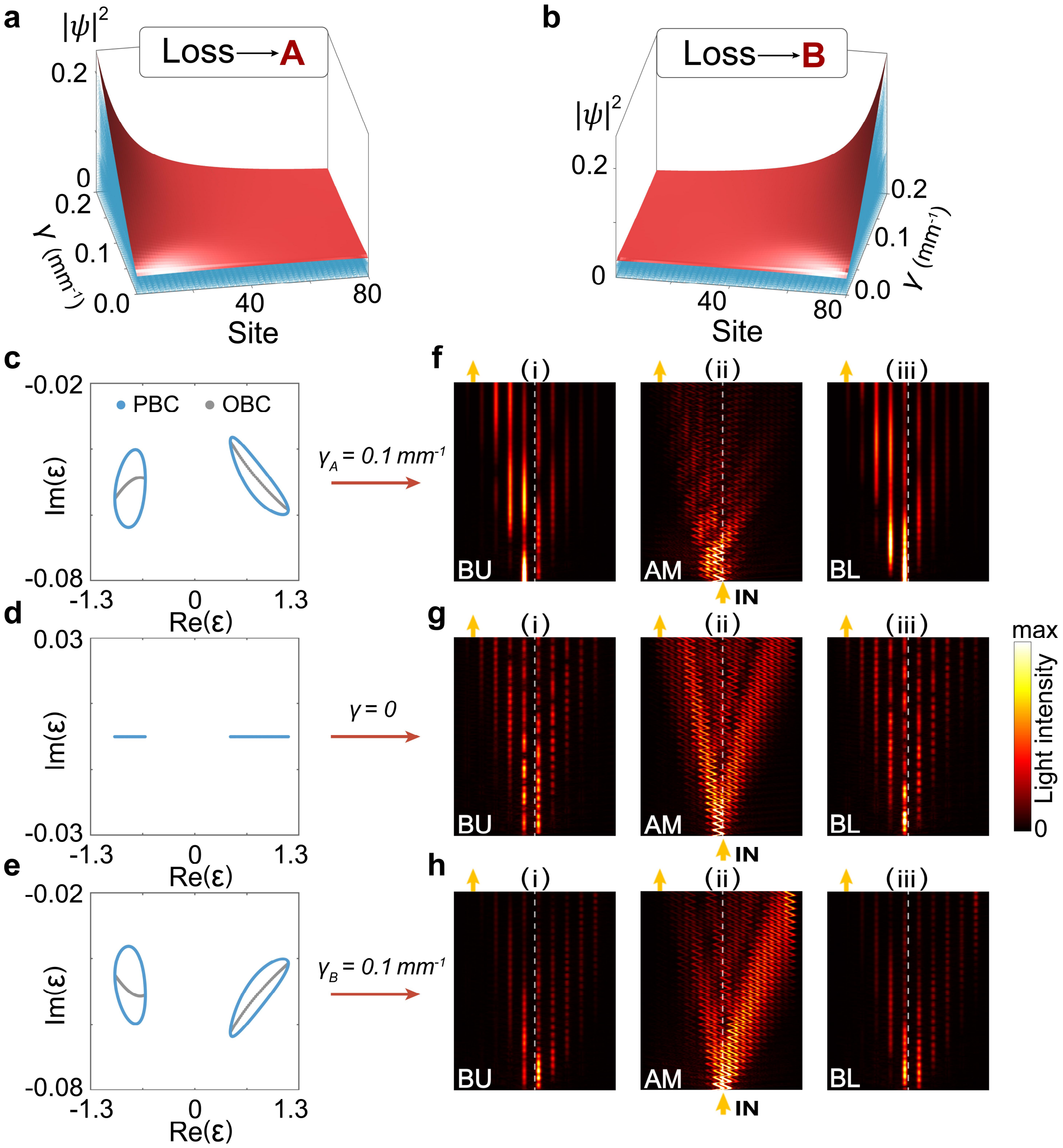

Fig. S7 a, b The eigenfunction distributions of the system with respect to the change of γ, where non-Hermitian losses are introduced into the sublattices A (**a**) and B (**b**). **c-e** The quasienergy spectra of the system corresponding to the cases of $\gamma_A = 0.1\ \mathrm{mm}^{-1}$ (**c**), $\gamma = 0$ (**d**) and $\gamma_B = 0.1\ \mathrm{mm}^{-1}$ (**e**), respectively. **f-h** Numerical simulations of light intensity distributions in the photonic system corresponding to the case in (**c**), (**d**) and (**e**), respectively.

Furthermore, we add the simulation results of the light intensity distributions corresponding to cases of $\gamma_A = 0.1\ \mathrm{mm}^{-1}$ (Fig. S7f), $\gamma = 0$ (Fig. S7g) and $\gamma_B = 0.1\ \mathrm{mm}^{-1}$ (Fig. S7h), respectively. These light dynamics intuitively demonstrate the pronounced influence of loss on the NHSE. Specifically, for the case with loss (Fig. S7f and 3h), a direct comparison with the results shown in Fig.

2a-d of the main text indicates that reducing the loss weakens the localization tendency of light. Correspondingly, when loss is entirely absent ($\gamma = 0$), the localization tendency vanishes (Fig. S7g).

Supplementary Note 5: Experimental details

(1) ***Sample fabrication and measurement***. The fabrication of photonic waveguides employed the technique of femtosecond laser direct writing. In experiment, we exploited a Ti:sapphire laser (Light Conversion Carbide 5W) to focus inside the boroaluminosilicate glass. To precisely control the movement speed of the glass, we utilized an Aerotech micro-displacement platform. This meticulous operation induced a slight change of refractive index (~0.004) in the focal region, successfully creating a single-mode parabolic photonic waveguide with a circular cross-section of about 6 μm in diameter. By finely adjusting the writing speed, we can fine-tune the effective modal index of the waveguide, achieving modulation of the propagation constant in the non-Hermitian Hamiltonian (see Fig. S8a). Furthermore, the coupling strength could be controlled by meticulously selecting the appropriate gap between adjacent waveguides, which is another key parameter in the non-Hermitian Hamiltonian (see Fig. S8b).

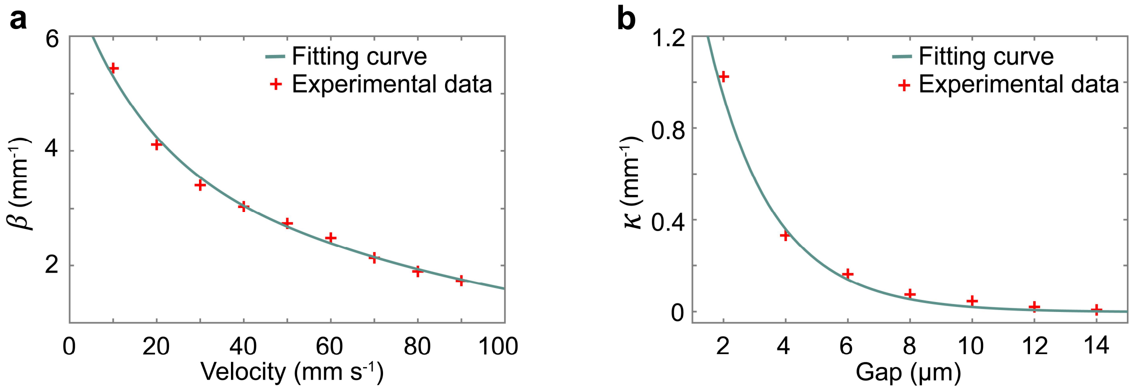

Fig. S8 a The propagation constant β as a function of the laser velocity. **b** The coupling strength κ as a function of the gap distance fitted from experimental data.

(2) ***Technique to introduce non-Hermitian losses.*** The on-site losses of the waveguide mode are effectively introduced through the application of a breaking line technique. Specifically, in the process of laser direct writing, the laser is periodically switched so that the waveguide is uniformly broken along the propagation direction. By controlling the turn-off time of the laser, the waveguide can be endowed with an adjustable loss. Fig. S9a shows the relationship between the loss coefficient γ and breaking length. We can see that the change of γ is more sensitive to the large breaking length, such that a smaller break length is selected to guarantee the accuracy of the loss in experiment. To introduce substantial loss in waveguide, we adopt a method involving the insertion of multiple breaking lines

with each period. In particular, we note that γ satisfies a linear relationship with respect to the breaking number (see Fig. S9b), which ensures the accuracy of the loss to a great extent.

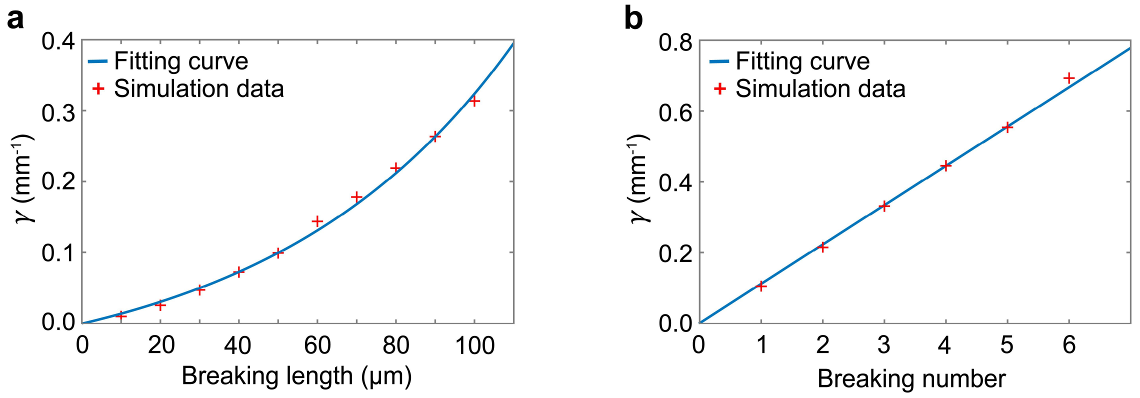

Fig. S9 The loss coefficient γ as a function of the breaking length (**a**) and breaking number (**b**) fitted from simulation data.

(3) ***The bending losses of the waveguides.*** The bending losses in photonic lattice experiments employing twisted waveguides are generally regarded as a critical experimental challenge. Fortunately, when the system operates within specific parameter regimes, these inherent bending losses are significantly lower than the on-site losses introduced into our system, and thus becomes negligible. For concreteness, we demonstrate the simulated bending loss dependence on waveguide structure parameters η and bending amplitude a in Fig. S10. We first consider the change of bending loss with η in the case of $a = 5.5$ μm, where we can see that the bending loss exhibits a significant change when $\eta < 0.25$. Once $\eta > 0.25$, however, the loss drops abruptly. Particularly within the parameter range of $\eta \geq 0.3$, the bending loss becomes far lower than the on-site loss ($\gamma_{A/B} = 0.2\ \text{mm}^{-1}$) introduced in the system. Furthermore, we present the change of bending loss with the bending amplitude a for the case of $\eta = 0.3$, where we observe the bending loss remains negligible over the full range of a. Consequently, in experiment, we selected the structural parameters $\eta = 0.3$ and $a = 5.5$ μm, corresponding to the star marker position in the figure. This configuration avoids the influence of asymmetric loss on the experimental results to the greatest extent, and ensures that the observed dynamic behavior and topological phenomena align closely with the predictions of our theory.

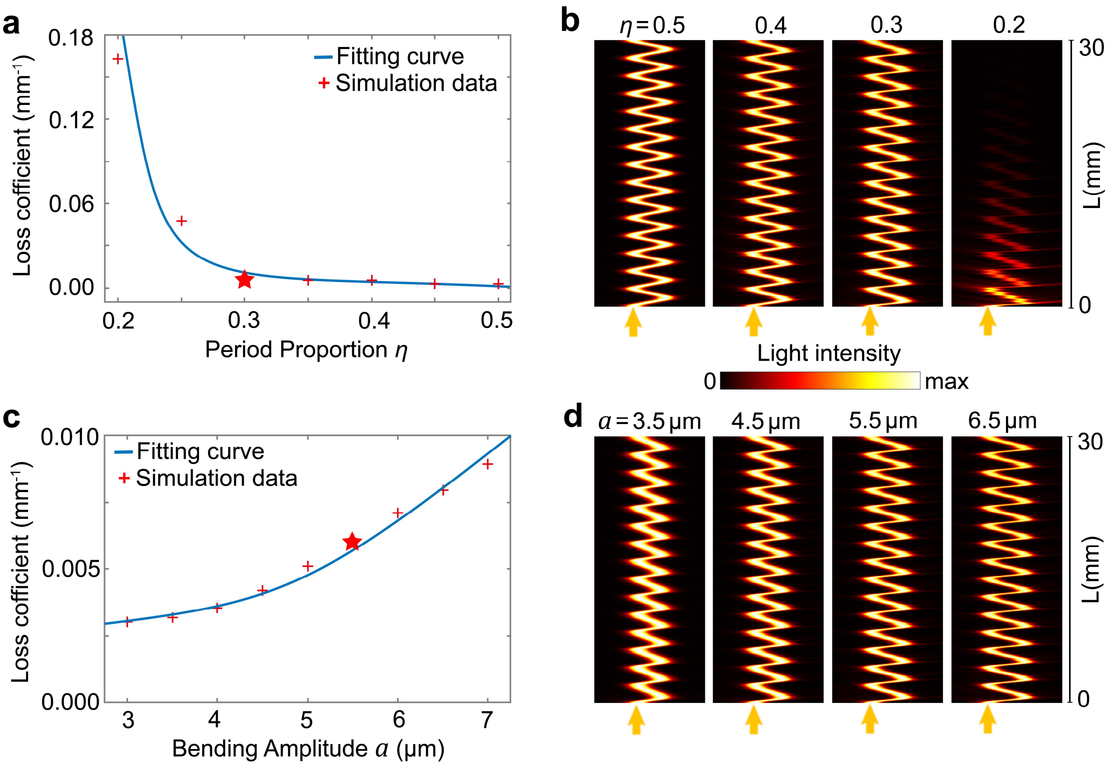

Fig. S10 a, b The bending loss coefficient γ at bending amplitude $a = 5.5$ μm for different period proportion η, and the corresponding simulation result. **c, d** The bending loss coefficient γ at $\eta = 0.3$ for different a, and the corresponding simulation result.

Supplementary Note 6: Origin of EPs in the coupled system

For the intercoupling of two 1D non-Hermitian chain with the same bending modulation as shown in Fig. 3a of the main text, each unit cell contains eight atoms. We have written the corresponding Bloch Hamiltonian in the main text as Eq. (2). According to the Floquet theory mentioned in Supplementary Note 2, we obtain an effective Hamiltonian H_{eff}^{c}. Considering the effect of on-site losses on the skin modes, the waveguide arrays LU and LD are endowed with different distributions of on-site losses so that two kinds of skin modes are introduced.

(1) ***EPs appearing in the momentum space.*** Here we set $\gamma_A^D = \gamma_B^U = 0$, while $\gamma_A^U = \gamma_B^D = 0.2\ \text{mm}^{-1}$. In the case of $t_c = 0$, there is no interplay between LU and LD, of which the eigenstates are localized in the opposite boundary, respectively. Fig. S11a shows the calculated complex quasienergy spectrum, where we find that the imaginary projections in momentum space corresponding to the LU and LD intersect at $k = k_0$ (see Fig. S11b), while the real projections are completely degenerate (see Fig. S11c). As t_c increases, multiply pairs of EPs appear, accompanied by the collapse of the quasienergy

spectrum, which has been discussed in the main text. Remarkably, each pair of EPs always appears at different k in momentum space. Regarding this phenomenon, we will provide an explanation in the following content.

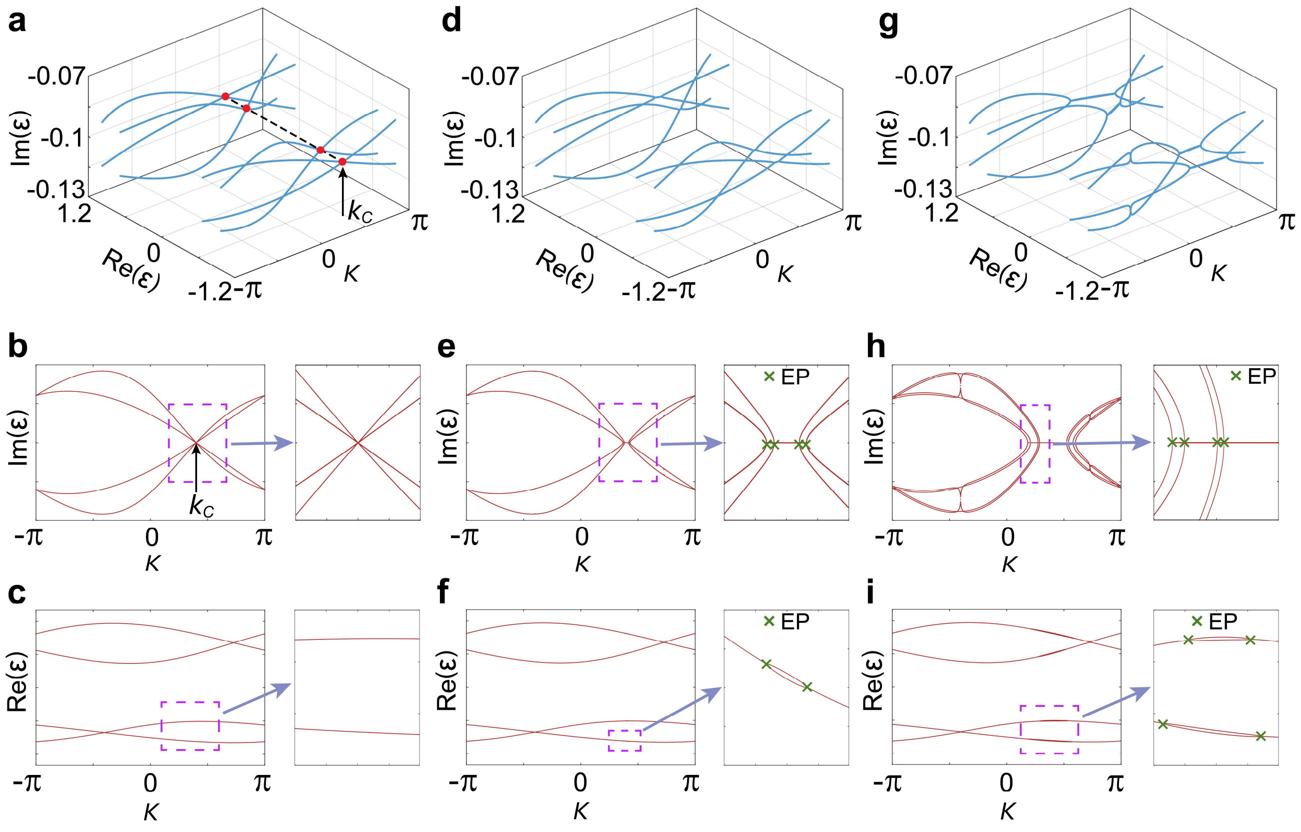

Fig. S11 The complex quasienergy spectrum and corresponding imaginary projection and real projection in momentum space for the case of $t_c = 0$ (**a**)-(**c**), $d_c = 20$ μm (**d**)-(**f**) and $d_c = 16$ μm (**g**)-(**i**).

We have mentioned in the main text that the appearance of any pair of EPs satisfies $|\gamma_1(k) - \gamma_2(k)| = 2t_c$, wherein the starting point of EPs satisfies $|\gamma_1(k_c) - \gamma_2(k_c)| = 0$. If t_c is sufficiently weak, the corresponding energy spectrum can be considered as depicted in Fig. S11a. We approximately regard $\gamma_{1/2}(k)$ as a linear change in the proximity of k_c, i.e., $\gamma_{1/2}(k) = \pm m(k - k_c)$, where m is the slope. The distance between any pair of EPs in the band structure of momentum space can be expressed as $D \propto t_c/m$. Figure S11d-f shows the quasienergy spectrum in momentum space when t_c is relatively weak, where we can see that the band with a larger slope always corresponds to a smaller D, which is consistent with expectation.

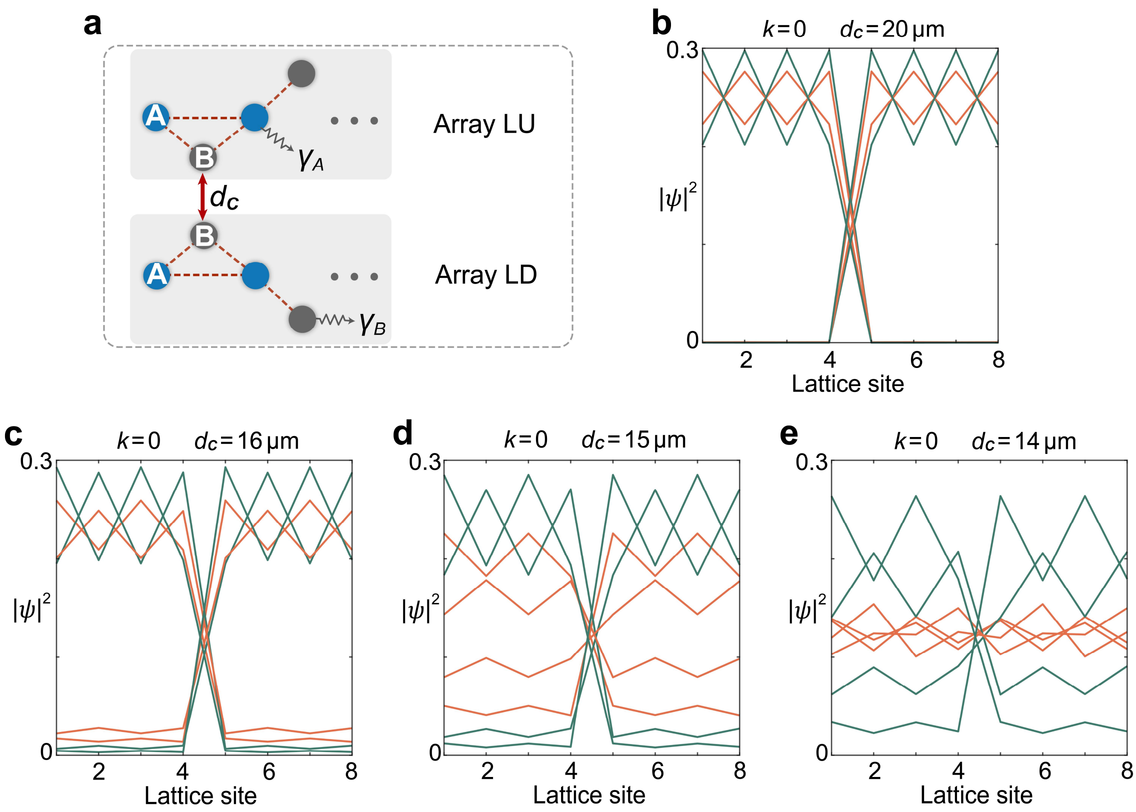

Fig. S12 **a** Schematics of the unit cell with eight sublattices. **b-e** The eigenstates calculated from the Bloch Hamiltonian with $d_c = 20$ μm (**b**), 16 μm (**c**), 15 μm (**d**) and 14 μm (**e**), where the Bloch momentum $k = 0$.

With the increasing of t_c, however, we note that the original coincident bands in imaginary projections are separated, resulting in different D (see Fig. S11g-i). Such result is due to the different eigenmode distribution of the momentum space in the lattice sites shown in Fig. S12a, resulting in the different t_c acting on each two-band pair. We show the eigenfunctions corresponding to $k = 0$ and $d_c = 20$ μm in Fig. S12b and find that the significant differences of the eigenmodes corresponding to the originally coincident bands in imaginary projections (marked by the same color), wherein the larger proportion of eigenmodes in sublattices B leads to larger t_c. To further illustrate this point, we show the distribution of eigenfunctions with $d_c = 16$ μm, 15 μm and 14 μm in Fig. S12c, S12d and S12e, respectively. With the decreasing of d_c, the eigenfunction corresponding to smaller m and larger t_C first exhibit the trend of degeneration, heralding the appearance of EPs.

(2) ***The absence of EPs.*** As shown in Fig. 3d, the EPs are absent when unequal losses are introduced into the photonic array. To clarify the phenomenon, we consider the case of $t_c = 0$ and $\Delta = 0.2$ mm^{-1}, and the corresponding quasienergy spectrum is shown in Fig. S13. One notes that the imaginary

projections in momentum space corresponding to the LU and LD no longer intersect at $k = k_c$ (see Fig. S13b), and the real projections are completely separated (see Fig. S13c). We rewrite the Eq. (2) in the main text as

$$H'(k) = \begin{bmatrix} \beta_1(k) - i\gamma_1(k) & t_c \\ t_c & \beta_2(k) - i\gamma_2(k) \end{bmatrix} \tag{S13}$$

where $\beta_1 \neq \beta_2$ for any k and $\gamma_1(k_c) \neq \gamma_2(k_c)$. As t_c increases, the corresponding eigenvalues are $E(k) = [\beta_1(k) + \beta_2(k)]/2 - i[\gamma_1(k) + \gamma_2(k)]/2 \pm \sqrt{t_c^2 + [\beta_1(k) - \beta_2(k) + i\gamma_2(k) - i\gamma_1(k)]^2/4}$, which no longer satisfies the condition of EPs.

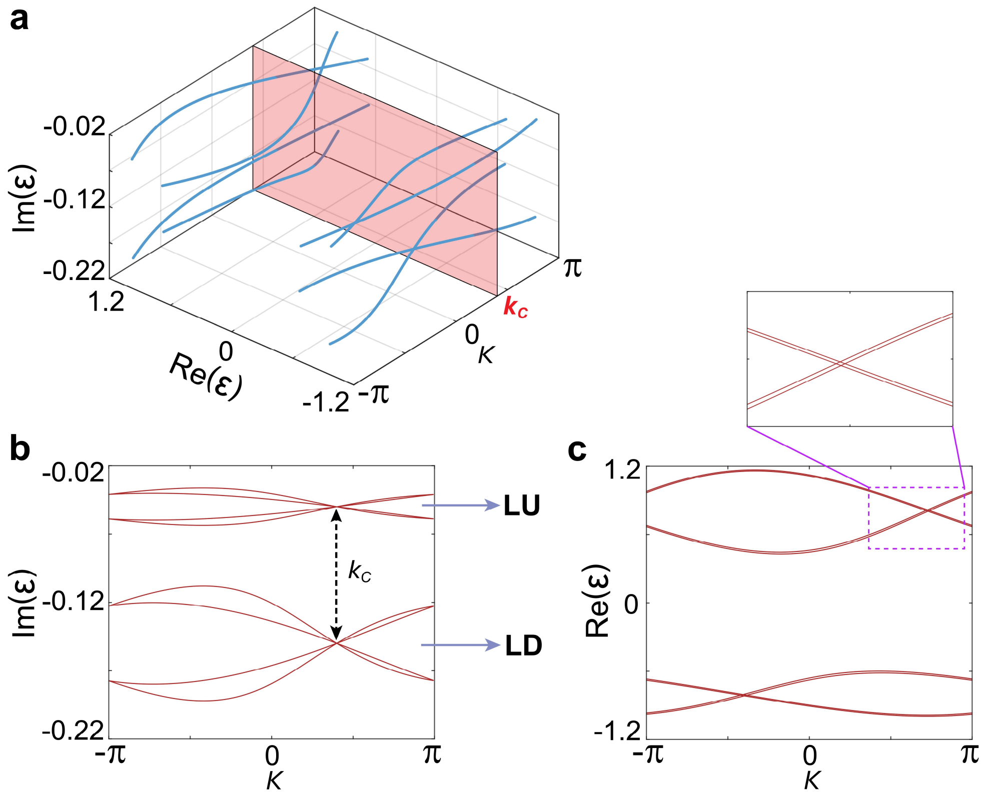

Fig. S13 The complex quasienergy spectrum (**a**) and corresponding imaginary projection (**b**) and real projection (**c**) in momentum space for the case of $t_c = 0$ and $\Delta = 0.2\ \mathrm{mm}^{-1}$.

(3) ***EPs appearing in the real space.*** In the previous sections, we have elucidated the emergency of EPs in the quasienergy spectrum under PBC. For the spectrum under OBC, however, the existence of EPs still merits further investigation, which would significantly strengthen our investigation into the role of EPs in modulating the NHSE.

Accordingly, we calculate the evolution of the spectrum under OBC in Fig. S14 and undertake a

detailed discussion. In the imaginary projection of the quasienergy spectrum shown in Fig. S14a, it can be observed that initially separated bands gradually approach each other as the coupling t_c increases, which culminates in the emergence of EPs at distinct positions, manifesting as degeneracies of different energy bands in the projection onto the imaginary axis $[Im(\varepsilon)]$. Correspondingly, the degenerate bands in the real projection are open at these EPs. Crucially, these EPs invariably arise from interactions between energy bands symmetrically distributed about $Im(\varepsilon) = i\gamma/2$, a signature inherited from the subsystem symmetry. Such phenomenon demonstrates that EPs in eigenvalue spectra under OBC originate from interactions between skin modes belonging to distinct subsystems. It is noted that these interacting skin modes correspond to eigenstates with opposing localization directions, while the emergency of EPs drives spatial overlap of these eigenstates, triggering a phase transition from boundary-localized to bulk-extended states. Thus, in our proposed coupled NHSE system, the emergence of EPs reveals the physical mechanism of delocalization, which is not only confirmed in the spectrum under OBC but also manifested in that under OBC.

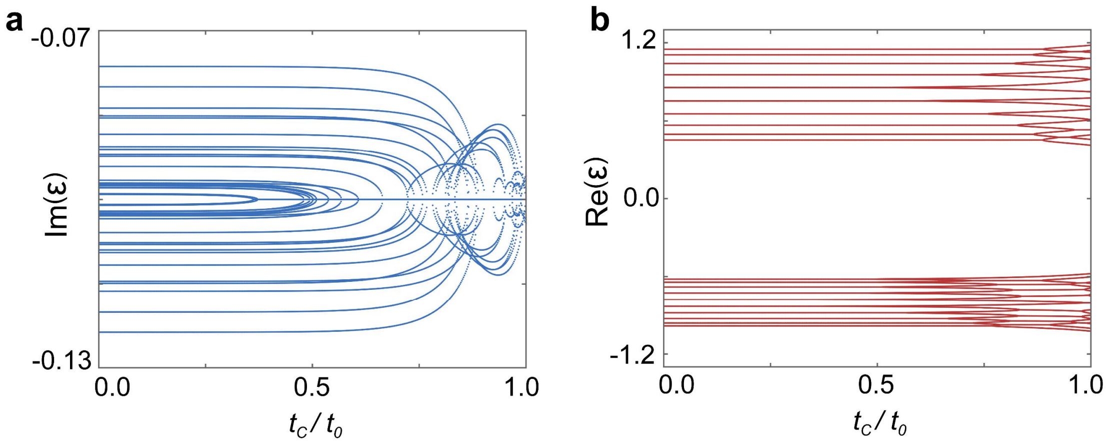

Fig. S14 The imaginary projection (**a**) and real projection (**b**) of the complex quasienergy spectrum under OBC for different t_c, where t_0 is the coupling strength corresponding to $d_c = 12$ μm.

Supplementary Note 7: The underlying PT-symmetry in coupled system.

For our proposed coupled system, an underlying PT-symmetry mechanism does indeed emerge. However, it is crucial to emphasize that the system is all-lossy and lacks additional gain. Thus, it can be interpreted as mapping an active PT-symmetric system involving both gain and loss onto an all-lossy system by introducing a global decay factor[17]. In the following, we will further elaborate that this emergence of PT-symmetry originates from the unique loss distribution and coupling configuration intrinsic to our system.

In the dual-lattice system illustrated in Fig. 3a of the main text, we emphasize that the two subsystems (the single-lattice arrays LU and LD) are identical in the absence of loss. Due to the

introduction of loss at different sublattices (A/B) within each subsystem, their respective eigenvalues spectra under periodic boundary condition (PBC) exhibit distinct topological properties, visually manifested by their opposite winding directions in momentum space. Correspondingly, when the losses are equal (i.e., $\Delta = 0$), the spectrum of the whole system symmetrically distributed about $Im(\varepsilon) = i\gamma/2$, as shown in Fig. 3b and 3c of the main text. Initially, with coupling $t_c = 0$, translational symmetry within each subsystem results in the formation of eight energy bands in momentum space within the eigenvalue spectrum, which can also be seen in Fig. S11a. This corresponds to the total number of sublattice sites per unit cell of the whole system. The spatial asymmetry of the loss distribution forces the real projection of the energy bands associated with the two subsystems to be degenerate, while their imaginary parts are symmetrically distributed about $Im(\varepsilon) = i\gamma/2$. In this case, the system can be considered effectively in a PT-broken phase. When $t_c \neq 0$, constrained by their individual translational symmetries, the inter-subsystem interaction predominantly occurs between corresponding sublattices – specifically, between sublattice i in subsystem LU and its counterpart sublattice in subsystem LD, which correspondingly ensures the translational symmetry of the whole system. Hereby, the spectral characteristics of the whole system can be analyzed by mapping them onto the interactions between pairs of corresponding bands. For this point, we introduced the two-level system mentioned in the main text to capture the spectral evolution with coupling t_c, that is

$$H'(k) = \begin{bmatrix} \beta_0(k) - i\gamma_1(k) & t_c \\ t_c & \beta_0(k) - i\gamma_2(k) \end{bmatrix} \tag{S14}$$

where $\gamma_1(k) + \gamma_2(k) = \gamma$, Defining $\gamma_{1/2}(k) = -\frac{i\gamma}{2} \pm i\gamma_\Delta(k)$, we rewrite the Hamiltonian as:

$$H'(k) = \begin{bmatrix} \beta_0(k) + i\gamma_\Delta(k) & t_c \\ t_c & \beta_0(k) - i\gamma_\Delta(k) \end{bmatrix} - \frac{i\gamma}{2} I \tag{S15}$$

Here we can see that the matrix $H_0' = \begin{bmatrix} \beta_0(k) + i\gamma_\Delta(k) & t_c \\ t_c & \beta_0(k) - i\gamma_\Delta(k) \end{bmatrix}$ exhibits PT symmetry. Thus, this two-level system can be equivalent as mapping an active PT-symmetric system onto an all-lossy system by introducing a global decay factor. As the coupling strength t_c increases, exceptional points (EPs) emerge at specific k-points satisfying the condition $|\gamma_1(k) - \gamma_2(k)| = |2\gamma_\Delta(k)| = 2t_c$. The emergence of these EPs signifies a phase transition from the PT-broken phase to the PT-exact phase, corresponding to the progressive coalescence of the imaginary parts of the energy bands at $Im(\varepsilon) = i\gamma/2$. This transition manifests spectrally as the collapse of the spectral area (the effective area

enclosed by the eigenvalues in the complex plane) under PBC. Ultimately, this process forces the skin modes to transform into bulk-extended modes, resulting in a delocalization within the system.

Therefore, this PT-symmetry mechanism represents a distinctive and essential feature unique to our coupled dual-lattice system, which also provides a strong foundation for both our theoretical framework and experimental results.

References

1. Zhang, L. et al. Acoustic non-Hermitian skin effect from twisted winding topology. *Nat. Commun.* **12,** 6297 (2021).
2. Zhang, X., Tian, Y., Jiang, J.-H., Lu, M.-H. & Chen, Y.-F. Observation of higher-order non-Hermitian skin effect. *Nat. Commun.* **12,** 5377 (2021).
3. Helbig, T. et al. Generalized bulk-boundary correspondence in non-Hermitian topolectrical circuits. *Nat. Phys.* **16,** 747 (2020).
4. Zou, D. et al. Observation of hybrid higher-order skin-topological effect in non-Hermitian topolectrical circuits. *Nat. Commun.* **12,** 7201 (2021).
5. Xiao, L. et al. Non-Hermitian bulk-boundary correspondence in quantum dynamics. *Nat. Phys.* **16,** 761 (2020).
6. Wang, W., Wang, X. & Ma, G. Non-Hermitian morphing of topological modes. *Nature* **608,** 50–55 (2022).
7. Li, Y., Lu, C., Zhang, S. & Liu, Y.-C. Loss-induced Floquet non-Hermitian skin effect. *Phys. Rev. B* **108,** L220301 (2023).
8. Ke, S. Wen, W., Zhao, D. & Wang, Y. Floquet engineering of the non-Hermitian skin effect in photonic waveguide arrays. *Phys. Rev. A* **107,** 053508 (2023).
9. Jiang, C. Liu, Y., Li, X., Song, Y. & Ke, S. Twist-induced non-Hermitian skin effect in optical waveguide arrays. *Appl. Phys. Lett.* **123,** 151101 (2023).
10. Sun, Y. et al. Photonic Floquet skin-topological effect. *Phys. Rev. Lett.* **132,** 063804 (2024).
11. Lin, Z. et al. Observation of Topological Transition in Floquet Non-Hermitian Skin Effects in Silicon Photonics. *Phys. Rev. Lett.* **133,** 073803 (2024).
12. Gong, Z. et al. Topological phases of non-Hermitian systems. *Phys. Rev. X* **8,** 031079 (2018).
13. Kawabata, K., Shiozaki, K., Ueda, M. & Sato, M. Symmetry and topology in non-Hermitian physics. *Phys. Rev. X* **9,** 041015 (2019).
14. Oka, T. & Kitamura, S. Floquet engineering of quantum materials. *Annu. Rev. Condens. Matter Phys.* **10,** 387 (2019).
15. Rodriguez-Vega, M., Vogl, M. & Fiete, G. A. Low-frequency and Moire-Floquet engineering: A review. *Ann. Phys. (Amsterdam)* **435,** 168434 (2021).
16. Eckardt, A. and Anisimovas, E. High-frequency approximation for periodically driven quantum systems from a Floquet-space perspective. *New J. Phys.* **17,** 093039 (2015).
17. Xia, S. et al. Nonlinear tuning of PT symmetry and non-Hermitian topological states. *Science* **372,** 72-76 (2021).
